# Quantification and Variability Analysis of Lignin Optical Properties for Colour-Dependent Industrial Applications

**DOI:** 10.3390/molecules23020377

**Published:** 2018-02-10

**Authors:** Olumoye Ajao, Jawad Jeaidi, Marzouk Benali, Andrea M. Restrepo, Naima El Mehdi, Yacine Boumghar

**Affiliations:** 1 Natural Resources Canada, CanmetENERGY, Varennes, QC J3X 1P7, Canada; olumoye.ajao@canada.ca (O.A.); jawad.jeaidi@canada.ca (J.J.); 2 Centre d’études des procédés chimiques du Québec (CEPROCQ), Montreal, QC H1N 1C1, Canada; arestrepo@cmaisonneuve.qc.ca (A.M.R.); nelmehdi@cmaisonneuve.qc.ca (N.E.M.); yboumghar@cmaisonneuve.qc.ca (Y.B.)

**Keywords:** lignin, colour, optical properties, biorefinery, carbon black, bioplastic, non-destructive measurement

## Abstract

Lignin availability has increased significantly due to the commercialization of several processes for recovery and further development of alternatives for integration into Kraft pulp mills. Also, progress in lignin characterization, understanding of its chemistry as well as processing methods have resulted in the identification of novel lignin-based products and potential derivatives, which can serve as building block chemicals. However, all these have not led to the successful commercialization of lignin-based chemicals and materials. This is because most analyses and characterizations focus only on the technical suitability and quantify only the composition, functional groups present, size and morphology. Optical properties, such as the colour, which influences the uptake by users for diverse applications, are neither taken into consideration nor analysed. This paper investigates the quantification of lignin optical properties and how they can be influenced by process operating conditions. Lignin extraction conditions were also successfully correlated to the powder colour. About 120 lignin samples were collected and the variability of their colours quantified with the CIE L*a*b* colour space. In addition, a robust and reproducible colour measurement method was developed. This work lays the foundation for identifying chromophore molecules in lignin, as a step towards correlating the colour to the functional groups and the purity.

## 1. Introduction

Lignin is the most abundant aromatic polymer in nature since it constitutes typically 18–35 wt% of lignocellulosic biomass, depending on the selected groups of species. It is spread around the outer surface of the cell wall in the middle lamella and is covalently linked with hemicelluloses. In plant stems, lignin plays the crucial role of conducting water in addition to providing rigidity [1,2] and it is hydrophobic [3]. Its polymeric structure is made of up to three aromatic alcohols (monolignols), known as *p*-coumaryl, coniferyl and sinapyl alcohol, whose aromatic parts are referred to as *p*-hydroxyphenyl (H), guaiacyl (G) and syringyl (S) units [4,5,6]. All biomass types exhibit differences (often significant) in their lignin content, the ratios of the monolignols and their interbonding patterns. As examples, agricultural lignins are composed of the three monomers, while softwood are made up of only the G and H sub-units, and hardwood lignins of G and S sub-units, respectively [7]. Lignin is to a large extent recovered from Kraft and sulphite pulping mills, and to a lesser extent from biorefineries. In spite of diverse properties and being sulphur-free, biorefinery lignins such as those obtained in the organolsov or enzymatic hydrolysis are still limited to niche markets and applications. In September 2015, the worldwide production of lignin was approximately 100 million tonnes/year, and its global market size was estimated at USD 732.7 million [8]. In the last decade, several lignin recovery processes came on stream while several others are still being developed or commercialized. The most recent industrial plants are all integrated with a Kraft pulping mill and include the LignoBoost^TM^ processes in 2013 (Plymouth, MA, USA) and 2015 (Kotka, Finland), as well as the LignoForce System^TM^ in 2016 (Hinton, AB, Canada).

Recent advances in lignin characterization and chemistry have led to the identification of innovative products and establishment of lignin as a promising renewable building block from the forest industry. This positioning of lignin is borne out of its cost-effectiveness, sustainability and ability to directly meet requirements for applications, or with modification in some cases. Although, lignin can be valorized into a wide range of chemicals and biomaterials, it is still underutilized when compared to cellulose [9]. Commercial scale development of lignin and its derivatives still requires evaluation and trade-offs between economics of scale, environmental impacts, products standards to facilitate trade, market trends and products aesthetics [10]. It is generally agreed upon, that lignin performance must be adequate and the cost competitive enough to displace petroleum-based alternatives. Some strategies for achieving this include lignin purification, depolymerization and functionalization, which can be done with organic-solvent-based fractionation, thermochemical processing, and catalytic processing [10,11]. Recovered lignin generally exhibits a complex amorphous and highly branched structure that depends on biomass species, harvest region and, more importantly, the extraction and recovery processes employed [12,13]. These differences have led to international collaboration for the development of standardized methods for lignin characterization that are reliable, harmonized and reproducible. It is envisaged that these methods will ensure the reliability, quality and judicious use of lignin by mitigating trade barriers and enabling the use and trade of lignin between producers and end-users in different organizations and countries. According to Jacobs et al. [14], the purity (carbohydrate content, ash, metals, sulphur, extractives and lignin content), molecular structure (molecular mass, molecular mass distribution, molecular size, functional groups) and thermal properties (glass transition temperature and decomposition temperature) are the most important lignin characteristics, but there are still no uniform or standardized methods. Currently, efforts are underway by scientists from organizations in forest resources rich countries to develop a series of ISO methods for seven groups of properties: (a) general composition (lignin, sugars, uronic acids, ash, solvent extractives); (b) functional groups (carboxyl, aliphatic and phenolic hydroxyl, methoxyl, aromatic groups); (c) size and morphology (molecular weight and particle size distributions); (d) thermal properties (glass transition, decomposition temperature, thermal stability); (e) structural features (mainly qualitative analysis); (f) safe handling and processability and (g) others (toxicity, skin/eye irritation, colour, solubility). Key contributors include VTT Technical Research Centre (Espoo, Finland), RISE Bioeconomy (Stockholm, Sweden) and FPInnovations (Pointe-Claire, QC, Canada), CanmetENERGY of Natural Resources Canada in collaboration with the Centre of Chemical Process Studies of Quebec (Montreal, QC, Canada), National Research Council Canada and the Canadian Standards Association.

Our review of the most common lignin characterization methods together with efforts to develop standards revealed that the colour of lignin is still often overlooked and rarely analyzed, even though both the colour and odour have long been recognized to limit the potential of lignin for use in diverse applications [15]. Colour limitation can be encountered irrespective of whether the lignin powder is utilized directly as recovered or not, made to undergo chemical modifications or thermal treatment prior to use. Specifically, it has also been noted that the use of dark colour lignins results in colour irregularities of the final product, as in the case of a polyurethane foam turning from yellow to brown upon lignin incorporation [16].

**Figure 1:**
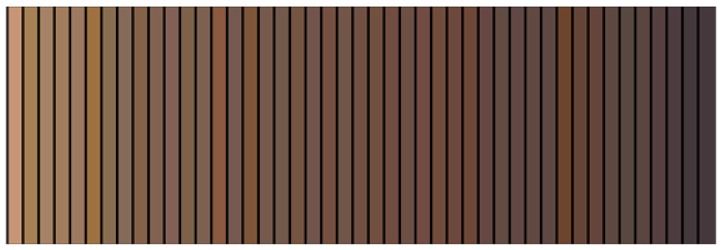
Measured colour for 45 lignin samples arranged in order of decreasing brightness.

The colour of 45 representative lignin samples out of the 120 that have been analysed are arranged in the order of decreasing brightness as shown in Figure 1. The samples originated from different biomass sources such as softwoods (32), hardwoods (5) and agricultural biomass (8). They can also be categorized by the different biomass fractionation methods that were employed during their extraction, namely: Kraft pulping (33), bisulfite pulping (1), dilute acid (7), ionic liquids (2), indigenous enzymatic hydrolysis (1), and industrial enzymatic hydrolysis of deconstructed wood (1). This illustrated colour range highlights the practical challenges associated with lignin colour and the research question this work answers.

**Figure 2:**
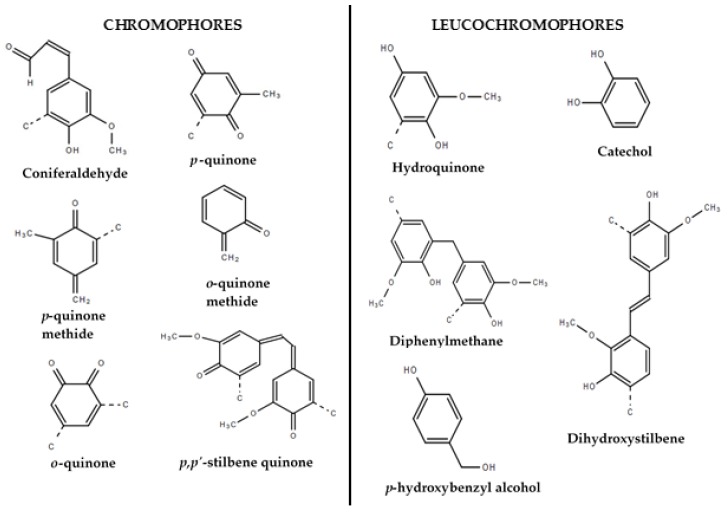
Some chromophores and leucochromphores from lignin.

The challenge with colour was recognized as far back as the early 1980s, when a U.S. patent was granted for a process of reduction of lignin colour, which described a two-step chemical process for blocking at least 90% of the free-phenoxyl and hydroxyl groups contained in a lignin, followed by an oxidation, to alter the colour of sulfonated alkali lignins and lignosulfonates [17]. Saritha et al. [18], also showed that native fungi can also be used to biologically decolourize lignin. A 2016 patent application for isolating and manipulating organosolv lignin stated that the colour of lignin cannot be masked in a cost-efficient manner with pigments, especially when light coloured material or products are desired, and this has been the basis for the research to obtain “light” or “white” lignin [19]. More recently, Zhang et al. [20] discovered that the fractionation of lignin by the use of methanol can lead to colour alteration. Since biomass fractionation, lignin extraction and recovery involve formation and/or elimination of multiple-bond functional groups, all unit operations of the lignin production processes have a direct impact on the colour. Although no quantification of the chromophores in lignin has been reported, chromophore formation and transformation during Kraft pulping has been reported by Lachenal et al. [21,22,23] and Dyer [24], but both were from a papermaking perspective and focused on bleaching of cellulosic fibers with low residual lignin content. In addition, it is presumed that the yellowness of lignin rich papers such as those from mechanical pulp, is due to the presence of chromophoric groups such as catechols, aromatic ketones, coniferaldehyde, stilbenes, and conjugated phenolics [25]. Lachenal and his peers have studied the coloured groups involved in Kraft pulp: chromophores arise from either carbohydrate or lignin. Their results suggest that pulp bleachability is greatly affected by the presence of lignin, especially in the form of quinones, which give a reddish hue [23]. Those derived from carbohydrates are easily destroyed by oxidation with conventional pulp bleaching chemicals such as chlorine dioxide. Dyer also reported that the main lignin-based chromophores include carbonyl functional groups, conjugated phenolics, quinoid structures and metal complexes. He also identified some leucochromophores (potential chromophores) that only become converted into chromophores upon oxidation. Figure 2 shows some of the chromophores and leucochrompohores that originate from lignin. A challenge for chromophore quantification is that it is not feasible to distinguish between all the chromophores present in a lignin sample. This is because lignin is a group of complex molecule with a varying number of functional groups and possible reaction sites are present in different ratios; the direct impacts of functional groups interactions as well as the effects of impurities within lignin (e.g., inorganic ones) on luminance have not yet been established.

We have screened a wide range of lignin derivatives and identified some products for which the colour can limit the use of lignin, such as paints, resins, plastics, binders, composite materials and cosmetics. As an example, lignin-based thermoplastic products are attractive because they combine wood appearance, thermoplastic advantages (e.g., hydrophobicity) and processability (e.g., extrusion and injection moulding). Lignins are also known to be UV-B absorbing compounds [26], and thus of potential use as a bio-component in cosmetic applications. Hence, their formulations are customized and optimized depending on the intended mechanical, thermal and optical properties. In addition, we have also identified areas where the optical properties can be used for process and quality control since colour and molecular structure are closely interrelated. Subsequently, a gap in the availability of standardized colour measurement methods and protocols was identified. A review of analytical methods for colour from either the pulp and paper or printing industry, shows that several standards and measurement protocols already exist. However, none of the existing methods focuses on redness so they all cannot be directly applied to lignin. For example, paper whiteness describes the ability to reflect all the wavelengths across the visible spectrum, whereas paper brightness measures the reflectance of a specific blue wavelength (457 nm). The main related standards come from the International Organization for Standardization (ISO) and the Technical Association of the Pulp & Paper Industry (TAPPI) [27,28,29,30,31]. At present, no standard or harmonized method for the analysis of lignin colour has been proposed in literature. The authors disclosed for the first time their preliminary research findings in the 7th Nordic Wood Biorefinery Conference held in Stockholm in 2017. 

This paper focusses on the optical properties of lignin, as it is a critical factor for development of markets, and the successful commercialization of some bio-products and materials requiring blending of lignin or thermochemical conversion. The specific objectives of this work are thus to:

- Develop, propose and validate a method for the quantitative analysis of lignin colour;
- Determine and document the variability of around the colour for various lignin;
- Identify the operating parameters that have significant influence on lignin colour;
- Assess the possibility and limitations of using the colour as a process control measure in lignin recovery and purification processes;
- Demonstrate how the optical properties can be used for controlling the production processes of various quality levels of lignin powders and their derivatives;
- Provide a suitable basis for the correlation of lignin chromophores and characteristics for future development of a simple, rapid, accurate and inexpensive analytical method.

## 2. Experimental

### 2.1. Materials

#### 2.1.1. Black Liquors

Two softwood black liquor samples (A and B) that had solids concentrations ranging from 46% to 49% were obtained from Canadian Kraft pulping mills located in the same geographical region of Eastern Canada. Liquor A had been subjected to an oxidative pre-treatment, an operation for decreasing the odour around a mill. Both liquors were diluted with ultra-pure water to 30% solids content, followed by pre-filtration using Grade 113 Whatman filter paper. The characteristics of the two liquors are given in Table 1. The characterization methods are described in Section 2.2.4. The values reported are averages of triplicate measurements. 

#### 2.1.2. Lignin Samples

The authors succeeded in collecting 120 different lignin samples from Europe and North America, which were screened. The received lignin originated from different commercial and emerging processes with technology readiness levels ranging from 5 to 9. The laboratory precipitated lignin samples were from the oxidized and non-oxidized black liquors described in Section 2.1.1. In total, the analysed lignin samples originated from softwood (110), hardwood (2), wheat straw (4), corn stover (2) and bamboo (2) as feedstock.

#### 2.1.3. Carbon Black Samples

Two groups of carbon black were studied by evaluating their optical properties. The first group of were generated from the carbonization of two Kraft lignins produced in a commercial scale mineral acid precipitation process and carbon dioxide precipitation processes, respectively. The second group comprised of two samples as well, a reference ASTM D 5098-Carbon Black and commercial carbon black.

### 2.2. Methods

**Figure 3:**
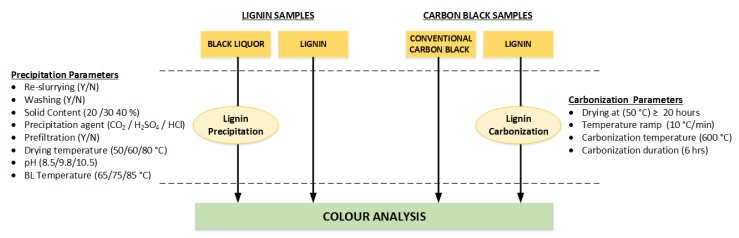
Samples and parameters evaluated for lignin characterization.

A diagram summarising the types of samples that were produced and analysed in this work is shown in Figure 3. To obtain lignin precipitated from black liquor under different conditions, several parameters were varied as listed in the diagram. This enabled direct comparison with technical lignin samples. It was also necessary to carbonize some lignin samples to benchmark against conventional carbon black. In a similar manner, the carbonization parameters are also provided.

#### 2.2.1. Experimental Setup and Procedure for Lignin Powder Production

**Figure 4:**
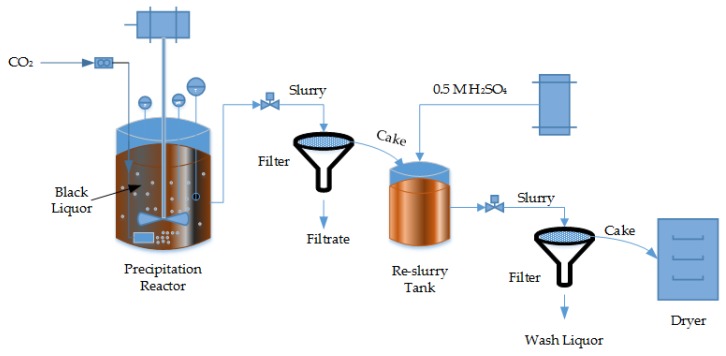
Schematic diagram of lab scale black liquor precipitation using carbon dioxide.

All the experiments were carried out in a closed and insulated 4.6-L vessel with standard baffles (T/10) and a cover plate. Heating was provided by an electrical jacket with controller connected to the outer jacket of the vessel. As shown in Figure 4, the carbon dioxide was introduced using a porous sparger tip located at the bottom of the vessel. The carbon dioxide flow rate was measured and adjusted using a rotameter placed between the sparger and the gas supply.

The key steps were similar to those reported by Kannangara et al. [32]. For all experiments, BL that had been diluted to a total solids level of 30% and thoroughly mixed is sampled for UV lignin content analysis. Subsequently, 2 L of the liquor is introduced into the reactor with the baffles in place, and mixed at 60 rpm using a two-stage, four-bladed, 45°-angle PBT with a 6.6 cm rotation diameter that is driven by an IKA-OST-20-Digital-Overhead-Stirrer with 1 rpm accuracy. Heating of the reactor starts simultaneously with mixing. After 75 °C is attained, about 3 mL of sample is withdrawn for pH sampling, and reinjected into the reactor, agitation is then increased to 120 rpm and CO_2_ injection at 1 L/min is commenced. Periodic pH sampling is continued at 5-min intervals and the CO_2_ sparging stopped when the pH is 9.8 ± 0.2. The agitation speed is reduced back to 60 rpm and allowed to run for 1 h to enable precipitation and coalescing of lignin and is known as the ageing step. This is followed by a cooling period during which the slurry is allowed to cool down to 55 °C with the agitation speed maintained. The slurry is then filtered in a 25 cm Buchner flask using a pre-weighed Whatman Grade 113 qualitative filter paper. The filtrate is collected for analysis of total solids, UV lignin content, ash content and viscosity as described in Section 2.2.4. The filter cakes obtained were either washed by adding 1.25 L of 0.4 M H_2_SO_4_ solution directly to the cake or adding the cake into a 4-L beaker containing the solution and stirring with a glass rod until it is free of clumps before filtration. For both cases, the washing step is completed by subsequently adding 250 mL onto the cake and re-filtration. The final filter cake obtained is placed in an aluminium pan; the mass is measured and then dried in an oven at 50 °C for up to 22 h and stopped when a constant mass is reached. The dried lignin is also analysed for the UV lignin and ash content. The colour of both cakes along the process and the dry lignin powder were determined as described in Section 2.2.3, with the complete protocol in Appendix A.

#### 2.2.2. Experimental Setup and Procedure for Carbon Black Production

**Figure 5:**
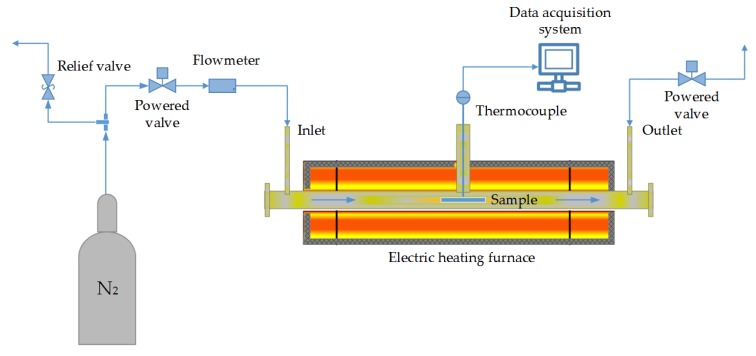
Experimental set-up for carbon black preparation.

Carbonization of lignin was carried out to evaluate the impact of thermal modification on the colour of an end product, i.e., carbon black. The experimental setup illustrated in Figure 5. The two Kraft lignin samples that were carbonized for benchmarking have been described in Section 2.1.3. Firstly, the samples were dried at 50 °C for at least 20 h until constant weight is attained. This was followed by placing about 200 g of each (the mineral acid precipitated Kraft lignin and CO_2_ precipitated Kraft lignin) in a laboratory muffle furnace where an inert atmosphere was maintained by a continuous flow of nitrogen. The temperature of the furnace was ramped up at 10 °C/min up to 600 °C and then maintained for 6 h before being allowed to cool and recover the thermal residue, which is carbon black.

#### 2.2.3. Colour Analysis by Spectrophotometry and Developed Protocol

**Figure 6:**
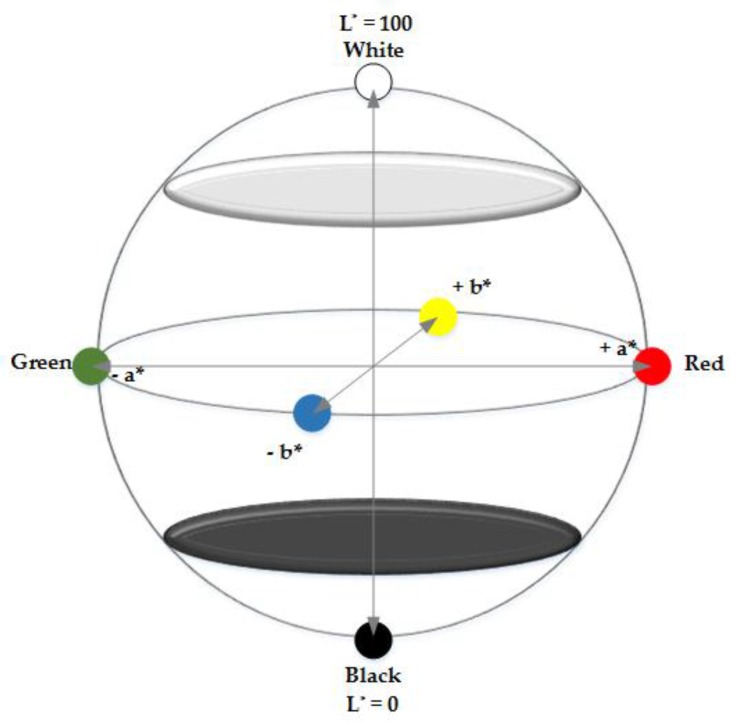
Illustration of the CIE L*a*b* colour space.

It was necessary to develop a colour measurement analytical method in the framework of this study as none had been reported for lignin. One of the objectives of this work was thus to disseminate this validated method with other researchers, engineers and bio-based product developers that are working at various stages of the lignin valorization chain, to enable communication of results and facilitate discussion. Hence, the detailed protocol is provided in the Appendix section of this paper. Briefly described, all the colour measurements were carried out in triplicate using a HunterLab ColorFlex EZ spectrophotometer that has a 45°/0° reflectance geometry. The standard illuminant (D65) defined by the International Commission on Illumination (CIE) was selected to simulate average daylight [33]. The standard CIE L*a*b* coordinates [34,35,36], as shown in Figure 6, were adopted and average values recorded for each sample as well as the standard deviations. 

The L* coordinate represents the psychometric index of lightness and ranges from 0 (black) to 100 (white); a* and b* corresponds to the chromaticity coordinates, with positions “−a*” (green), “+a*” (red), “−b*” (blue) and “+b*” (yellow) respectively. The variation in colours between a reference colour and each measurement were quantified from the coordinates with the CIEDE2000 (Δ*E*_00_) formula given in Equation (1), with the parametric weighting factors as given by in the ISO standard and Sharma et al. [37,38]. This equation involves an intermediate conversion to L*C*h coordinates, and Δ*L’* is the difference in lightness and darkness, Δ*C’* is the difference in chroma (+ implies brighter and − duller) and Δ*H’* is the difference in hue. Positional corrections were also applied for the lack of uniformity with the CIE L*a*b* as lightness compensation *(S_L_*), chroma compensation (*S_C_*), hue compensation (*S_H_*), as well as parametric factors *k_L_, k_C_, k_H_* to correctly account for the influence of experimental viewing conditions [39]; *R_T_* represents the spectral reflectance factor.

(1) $$\Delta E_{00}=\sqrt{{\left(\frac{\Delta L^{\prime }}{k_{L}S_{L}}\right)}^{2}+\;{\left(\frac{\Delta C^{\prime }}{k_{c}S_{c}}\right)}^{2}+\;{\left(\frac{\Delta H^{\prime }}{k_{H}S_{H}}\right)}^{2}+R_{T}\frac{\Delta C^{\prime }}{k_{C}S_{C}}\frac{\Delta H^{\prime }}{k_{H}S_{H}}}$$

In addition, the Browning Index (*BI*) for all lignin samples was quantified via Equation (2):

(2) $$BI=100\times \left(\frac{X-0.31}{0.17}\right),$$

where:

(3) $$X=\frac{\left(a^{*}+1.75L^{*}\right)a^{*}}{\left(5.645L^{*}+{\;a}^{*}-3.012b^{*}\right)}.$$

#### 2.2.4. Other Analysis

The total solid content for the black liquors and lignin powders were determined by drying a given amount of material at 105 °C according to the CPPA J.15 (II) standard test procedure [40]. The UV lignin content was determined at a wavelength of 280 nm using a Shimadzu UV Visible Spectrophotometer, model UV-1700 spectrophotometer. About 1 g of black liquor or lignin was dissolved in 100 mL of 0.1 M NaOH, and was diluted until the absorbance at 280 nm was in the 0.3–0.8 range for an absorption coefficient of 23.7 dm^3^g^−1^cm^−1^. 

The Klason lignin content, acid soluble lignin and carbohydrate content were determined based on a protocol adapted from the Innventia Biorefinery Test Methods L2:2016 (Stockholm, Sweden) developed for Kraft lignins. In this method, 3 mL of 72% wt/wt sulfuric acid was added to a 200 mg sample of oven-dried black liquor or 300 mg of lignin. The samples were placed in a water bath at 30 °C for 1 h, stirred, diluted with deionized water and hydrolyzed at 120 ± 1 °C in an autoclave. Subsequently, the samples were filtered to recover the acid insoluble (Klason) lignin, and the weight was determined. The filtrate was diluted by a factor of 1/100 and the acid soluble lignin content was determined using the same Shimadzu UV Visible Spectrophotometer but at a wavelength of 205 nm using an absorption coefficient of 110 dm^3^g^−1^cm^−1^ while the carbohydrate content was determined by high-performance liquid chromatography (HPLC). The ash content of the black liquor was determined by heating a known quantity of residue from total solids content determination at 950 °C for 20 h, a method adapted from Holmqvist [41]. The inorganic residue in the samples, which is referred to ash were measured as described by Holmqvist and Wallberg [41,42]. The residual effective alkali content was determined by capillary electrophoresis with direct UV-detection based on the recommendations reported by Radiotis et al. [43]. The viscosity measurements for the black liquors were performed with a Brookfield LVDV-II+Pro viscometer (Oakville, ON, Canada) at room temperature.

## 3. Results and Discussions

### 3.1. Lignin Colour Variability and Identification of Influencing Parameters

In general, lignin precipitation can be achieved by adjusting pH and ionic forces. In alkaline solution, lignin molecules are negatively charged as a result of hydroxyl and carboxyl groups that are dissociated. The lignin is stable in aqueous solution due to the repulsive electrostatic forces. When the pH is lowered by acidification, protons (H^+^) counteract the negatively charged groups and reduce the force of repulsion in the process, leading to instability of the molecules and therefore precipitation. To determine the parameters that can be varied in order to manipulate lignin colour during the precipitation and recovery process, a list comprising 22 variables was identified for the precipitation, washing and drying step 6. They were then categorized into 13 independent (Temperature, CO_2_ or acid concentration, CO_2_ purity, hemicellulose content, agitator speed and power mixing condition, pressure, black liquor sampling withdrawal, solids concentration, residence time, and slurry conditioning) and 9 dependent variables (pH, gas emissions, molecular weight distribution, filtration resistance, structure of particles, lignin purity, low residue content lignin composition, high residue content lignin composition). The list were further refined into a shorter list of factors that would have an effect on the economics and feasibility of operating a lignin precipitation process. The shortlisted variables found in this approach were those identified to have the greatest impact on the economic feasibility of lignin precipitation by acidification and recovery from black liquors. They include the pH, temperature (°C), solid content (%), the presence of prefiltration (yes/no), washing intensity (L of washing liquor per kg of lignin) and drying temperature (°C). A reference precipitation case was defined and the colour changes due to variation were benchmarked against these. The results for each of the respective parameters are presented in Section 3.2, Section 3.3 and Section 3.4. 

**Figure 7:**
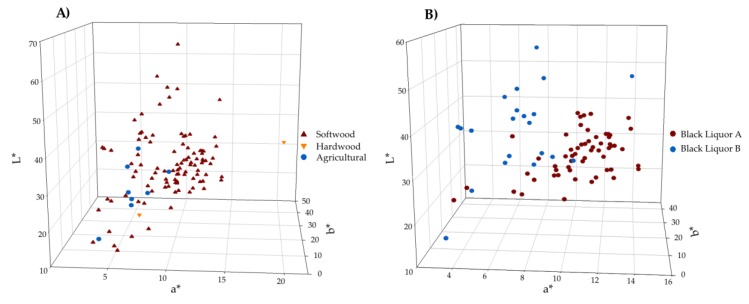
Lignin colour variability depending on (**A**) feedstock and types of (**B**) black liquors.

As illustrated by Figure 7, there is a large variability in lignin colour whether it is for Kraft lignin precipitated from black liquors or lignin resulting from other processes. More specifically, the feedstock-based colour spaces overlap as shown in Figure 7A, where the 120 lignins were plotted. Considering an application that requires a narrow range of colour specifications, it would thus be necessary to optimize the lignin recovery process for the required colour. It also appears that the single hardwood lignin sample from organosolv fractionation process is an outlier but there was not enough data to give a clear confirmation of this interpretation. For determining the impact of operating conditions on the colour of the black liquor-based lignin, the colour of samples precipitated under identical operating conditions were plotted for softwood Kraft mills A and B. The black liquor from mill A is oxidized and contained less of acid soluble lignin (57 vs. 75 mg/g), and total organic carbon content (185 vs. 226 g/L) compared to that from mill B. Also the residual alkali for mill A is lower (2.31 vs. 23.26 g/L as Na_2_O). As shown in Figure 7B, both data sets differ mainly with respect to the a* dimension, which mean that mill B lignins exhibit a lower degree of redness. Therefore, this could be due to the presence of leucochromophores (potential chromophores) that are converted into chromophores upon oxidation. As specific examples, several studies have reported that catechol structures are formed during Kraft pulping [24,44], due to demethylation reactions. Catechol is one of the main leucochromophores that is oxidized into *o*-quinone, which is a chromophore (both illustrated in Figure 2).

As there is no reference lignin colour, it was necessary to select one colour with which the Δ*E*_00_ was calculated. The reference lignin was a commercial Kraft lignin with CIE L*a*b* values of (29; 12; 17). The calculated Δ*E*_00_ values for all the samples ranged between 1.4 and 36.6, which means that each lignin has a differentiable colour since the human eye is able to discriminate colours when Δ*E*_00_ is higher than 1. The colour variability has also been evaluated in terms of the browning index, which ranged from −107 to 7969. About 3% of the 120 samples had negative values, which implies that this index that was developed for use in the food industry is not generally suitable for all lignins.

### 3.2. Effect of Precipitation and Drying Temperature on Lignin Colour

**Figure 8:**
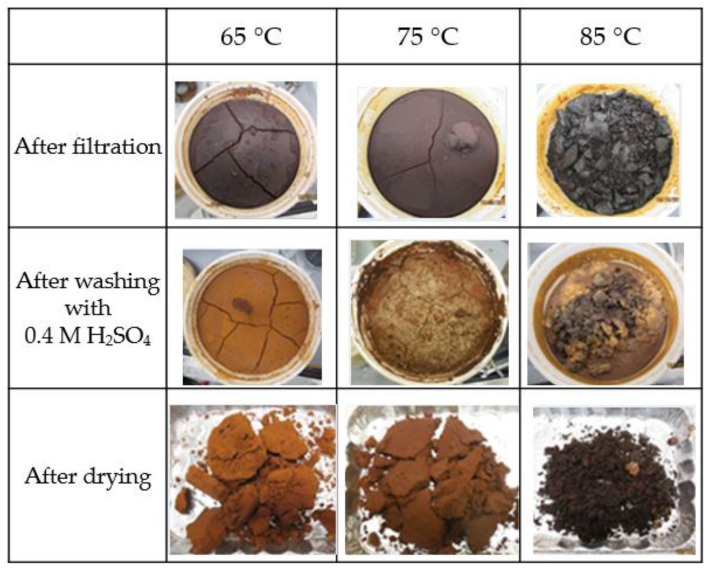
Contrast in lignin colour when precipitated at different 65, 75 and 85 °C.

Varying the temperature for lignin precipitation within the reported range for successful precipitation (65–85 °C) had two main effects as shown in Figure 8. Firstly, a clear colour difference was observed by increasing the precipitation temperature. The colour of the cake obtained after filtration was darkest for 85 °C and the trend persisted even after washing with 1.25 L of 0.4 M H_2_SO_4_ followed 250 mL of distilled water. The same observation was made after the cake had been dried. Specifically, for black liquor A, the dry lignin that was precipitated at 65 °C had CIE L*a*b* reference values of 43; 14; 26, and a Δ*E*_00_ of 7.6 and 5.6 when compared to those at 75 °C and 85 °C respectively. This nonlinear trend is due to the L coordinate, which had a minimum at 75 °C. An identical trend was observed for black liquor B as well. Specifically, a lower temperature also led to a higher lignin precipitation yield for both black liquors and is in agreement with a previous study by Evstigneev [45] that showed the solubility of lignin increases with temperature, and is due to a lower dissociation constant (K_a_) and the consequent increased solubility exhibited by lignin. Additionally, it is known that the higher the lignin yield, the higher the amount of lower molecular weight lignin fractions that are precipitated [46]. Therefore, the colour changes can be can be attributed to the alteration of existing chromophores, formation of new ones or changes in chromophores composition. Recent work by Li et al. [47] on optical property changes of enzymatic hydrolysis lignin has also confirmed that a positive correlation exists between lignin colour and increasing temperature. The changes are either due to change of the lignin structure, or fragmentation of the molecules and thus the chromophores. Any of such changes also changes the ratio of chromophores and ultimately the observed colours. The second observed effect, the formation of soft and sticky large clumps that were difficult to break down, also substantiated the previous observation because the flowability of lignin increases as the glass transition temperature is exceeded, and this leads to faster mass transfer and condensation reaction kinetics. An important inference that we can make is that the condensation reactions are also one of the factors that play an important role in chromophore changes. This is because condensation reactions are known to increase the likelihood of conjugated structure.

A temperature effect was also observed when drying lignin. Preliminary tests were carried out by drying lignin that had been precipitated from black liquor. Visual inspection of the dried sample revealed a stark contrast between samples dried at temperatures of 50 °C and 105 °C. It was therefore necessary to determine the recommended drying temperature to maintain colour and the tolerance limits that must be ensured. Some samples were transformed into dark clumps without any resemblance to the starting material. We also found out that a significant colour difference is observed if the variation of drying temperature exceeds ±5 °C. Based on the visual observations and confirmation with the CIE L*a*b* values, it can be concluded that the temperature is a critical operating parameter that influences the colour of lignin at the precipitation as well as the drying steps. 

### 3.3. Effect of Precipitation Agent and pH on Lignin Colour

**Figure 9:**
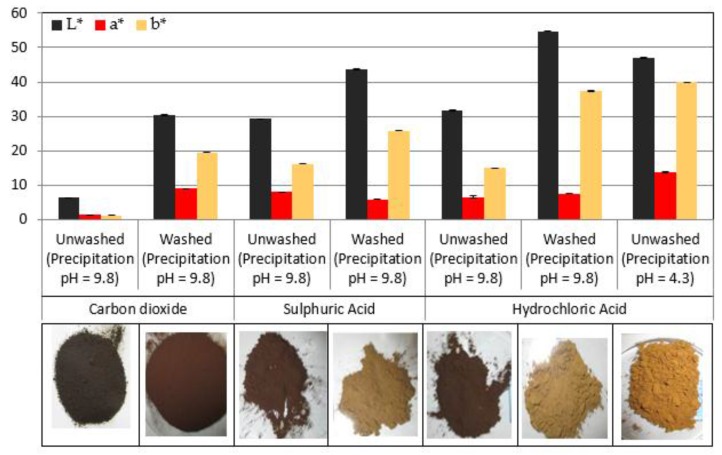
Effect of precipitation agent and pH on lignin colour.

Three different precipitation agents were investigated in this study, namely carbon dioxide (CO_2_), hydrochloric (HCl) and sulfuric (H_2_SO_4_) acids. CO_2_ is the acidification agent employed in most recent commercial lignin precipitation processes, whereas sulfuric acid is used in conventional Kraft lignin such as Indulin. Although hydrochloric acid is not in use for Kraft pulp mills integrated processes because of the presence of chlorine (a non-process element), it has been used in several fundamental studies on lignin precipitation. They all lower the pH of black liquor by neutralizing the hydroxyl groups. Consequently, protonation of the charged groups on the lignin molecules occurs and this induces precipitation. The differences in colour are allegedly partly due to the difference in acid strengths and due to their acid dissociation constants, as indicated by their pKa at 25 °C. The injection of CO_2_ into black liquor results in the formation of carbonic acid, a weak acid (with two pKa values of 6.4 and 10.3), which is a significantly weaker than either the strong acid HCl (pKa = −7) and the strong acid H_2_SO_4_ (pKa = −3 and pKa = 2). Having different proton dissociation constant implies different proton concentration and thus an impact on the thermodynamic equilibrium of the reaction. In addition, the kinetics of the protonation reaction is significantly slower for the gaseous carbon dioxide compared to the liquid acidification agents as there is diffusion gas in the black liquor. Therefore, it is expected that different degrees of protonation would occur with the different acidification agents. The content of functional groups such as hydroxyl and methoxyl is consequently influenced by the type of acid employed [48,49]. Lignin polymers are difficult to purify and characterize because of the highly heterogeneous linkages that occur between subunits within lignin and linkages to polysaccharides. Therefore, lignin contains several inter-molecular linkages, and the β–O–4 is one of the most dominant making up 40–60% of all linkages in wood [13]. Exposure to mild acidic conditions lead to a cleavage of some of the bonds (acidolysis and hydrolysis), and it has been shown by Santos et al. [50], that the rate of such reactions is higher for HCl than H_2_SO_4_. Additionally, it was also reported that the predominant reaction routes were different for both acids. Due to all these reasons, it can be expected to have different amounts of conjugated structures, which leads to the different colours observed and illustrated in Figure 9. Another contributing factor is the difference in ionic forces exhibited by Cl^−^ and ${\mathrm{SO}}_{4}^{2-}$. This means that the precipitated lignin would have differences in chemical composition. 

A decrease of the precipitation pH led to an increase of precipitation yield for both studied black liquors, which means that more of the negatively charged lignin molecules were protonated and precipitated. Therefore, it is possible to deduce that the increase in the brightness of the lignin with decreasing pH is due to a difference in the relative proportions of conjugated groups that were precipitated. It is expected that the groups will include most of the dissolved lignin chromophoric meoieties such as quinone methides, quinone, catechols, hydroquinone and cinamyl aldehydes [20,47,51].

### 3.4. Correlation of Lignin Colour Properties with Precipitation Conditions

**Figure 10:**
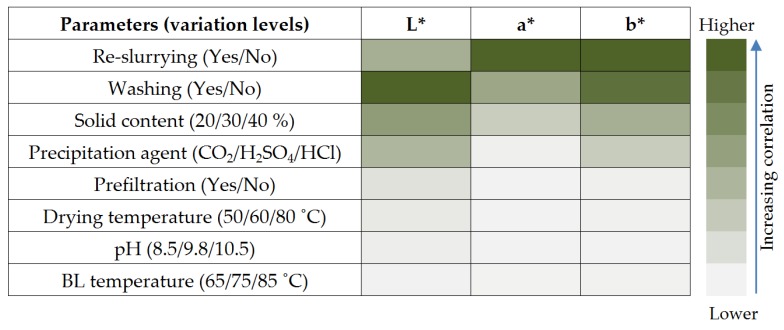
Correlation lignin colour properties to precipitation conditions.

A correlation between the changes observed for the different lignins and the factors that have the strongest influence was developed in order to demonstrate the concept that the lignin recovery process can be monitored and controlled using colour measurement. Such a correlation also enables to determine the best strategy for manipulating the lignin colour according to a specific application. This was done by evaluating and quantifying the variations observed in terms of the CIE L*a*b* coordinates. It has to be stressed in this regard that visible colour differences by human observation sometimes do not imply a large shift on the coordinates. The correlation of all the varied conditions with colour is illustrated in Figure 10. The acid-based re-slurrying of the lignin cake during the washing step had the most significant impact on the lignin colour, specifically the blueness (a*) and yellowness (b*) of the samples. This impact was more pronounced than when washed by vacuum filtration. This can be explained by the faster kinetics of reaction. During washing at a pH typically between 1 and 4, protonation of the lignin occurs and the bounded sodium is substituted. In comparison to an unwashed lignin, a brighter lignin is obtained due to the dilution of the cake and/or the displacement of the black liquor that is trapped in the cake. This explains why there is a significant impact on the darkness (L*) of the samples obtained. Washing efficiency and the colour obtained will depend, to an extent, on the shape, porosity gradient and distribution of the pore size. The solid content and the precipitation agent could also be correlated. 

This correlation was used to determine and map in what steps and locations of a lignin precipitation and recovery process, the colour can be employed as a process control tool. Four locations where colour-based control strategies can be implemented were identified to be the: (i) coagulation reactor exit; (ii) filter press exit; (iii) exit of the second filter press after re-slurrying; and (iv) dryer exit. It should also be noted that this correlation do not cover synergistic effects when several variables are changed simultaneously, hence extension to such studies in the future is considered necessary for developing predictive models and soft sensor controllers. 

### 3.5. Carbon Black Application Case Study

Lignin derived carbon black substitute has been proposed as a promising derivative because its production is not an energy and GHG intensive process compared to others and it can be used in a wide range of applications. Potential uses include: tires and industrial rubber products, printing inks, plastics, high performance coatings, electrostatic discharge compounds, composites, thermal paste, and thermally conductive fillers. To meet the requirements of carbon black applications, some of the sought after and evaluated properties include: thermal stability, UV absorbance, specific surface area, particle size, molecular structure and electrical conductivity. Despite the number of reported studies on lignin, the colour of the carbonized lignin is rarely measured or discussed, even though it can limit its use. The importance of colour is underscored by the fact that colour indicators, which include the tint strength, lightfastness rating, colour index, the International Commission on Illumination CIE L*a*b* colour space as well as the Munsell Notation Listing (Hue black, Value and Chroma), are used for describing the grades and suitability of carbon black. In addition, the L* and b* are essentially used to indicate the jetness (blackness) and undertones of carbon black, because a sample with a lower b* reading would appear to be more blue than another sample with identical L* values. Carbonized lignin was prepared as described in Section 3.3 to enable comparison with a commercial and reference sample of known specifications. The results of the comparison are shown in Table 2. The ASTM D 5098 conforming carbon black was used as the benchmark, against which all the others were compared. The colours of the two commercial lignins were significantly different to that of carbon black (Δ*E*_00_ of 30.09 and 15.98, respectively). The effect of carbonization is seen in the reduction of the L*, a* and b* values. A careful selection of the operating conditions led to carbonized lignin with the colour closer to the reference but nevertheless significantly different (Δ*E*_00_ of 2.9 and 2.3, respectively). Based on these results, it is possible to conclude that lignin derivatives that require carbonization are less sensitive to initial lignin colour. In addition, with optimized carbonization conditions, a carbon black substitute with suitable colour specifications can be obtained. However, since the yield is inversely proportional to the darkness, a trade-off between both factors will always be required.

## 4. Future Research on Lignin Colour and Knowledge Gaps to be Addressed

**Figure 11:**
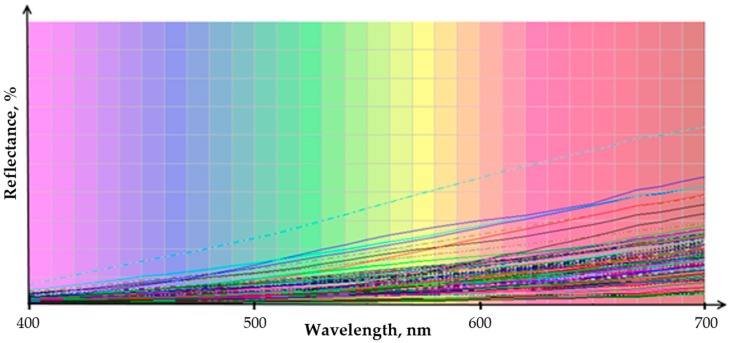
Reflectance spectrum for the lignin samples.

Two key challenges associated with lignin colour have been identified in this study and need to be addressed in future works. Firstly, there is no reference lignin standard of acceptable colour that can be used as a benchmarking standard for a colour index development. The lignin scientific community and manufacturers must agree and define a reference lignin or application requirement colour. The defined standard must be globally acceptable as a representative model that facilitates most high-value specialized industrial applications. Furthermore, the browning index that has already been established in the food processing industry was used in this work, but we consider it necessary that a more specific lignin colour index will be developed in the future. Preliminary results on the suitability of the proposed approach led us to conclude that it must take into account the reflectance over a wide range of wavelengths. A comparison of 120 different lignin samples was carried out. They had differences in feedstock (i.e., softwood, hardwood, wheat straw, bamboo, corn stover), fractionation methods (e.g., Kraft pulping, soda, bisulphite, organosolv, ionic liquid, supercritical CO_2_, etc.) and recovery methods (e.g., enzymatic hydrolysis, supercritical H_2_O, mineral acid and electrochemical acidification). The most divergent reflectance trend can be seen at wavelengths between 620 and 700 nm, as shown in Figure 11, and this means that the main differences between the lignins is the degree of redness. Therefore, to develop a colour index that is sensitive to lignin colour differences, the CIE L*a*b* coordinates corresponding to the wavelength range must be considered. 

In the future, we will also try to enhance the understanding of the dominant factors that influence the colour of recovered lignin at a molecular level. We recognize that a direct correlation of colour to the functional groups present in a lignin sample is challenging because functional groups alone do not necessarily induce colour. They only do when they are parts of chromophoric molecules with specific conjugated chemical systems that are able to absorb a photon of specific energy levels corresponding to the visible light spectrum (wavelengths: 400–750 nm), as described by the Equation (4). From the perspective of quantum chemistry of molecules, conjugated systems exhibit delocalized electrons across the multiple p orbitals involved in π type (double) bonds. Typically, conjugated systems have long carbon chains of alternating single and double covalent bonds and eventually atoms with unshared electron pair. An inverse relationship exists between energy and wavelength as shown in Equation (4), where *E* is the photon energy, *h* is the Planck constant, *c* is the speed of light in vacuum and *λ* is the photon’s wavelength:

(4) $$E=\frac{h\;c}{\lambda }.$$

When visible light (photons) strikes a chromophore, a portion is absorbed relative to the differences in energy levels between the Highest Occupied Molecular Orbital and the Lowest Unoccupied Molecular Orbital (HOMO-LUMO gap). This gap is unique for each molecule and depends on the degree of conjugation. The unabsorbed fraction is reflected as the complementary colour for the absorbed wavelengths. Another phenomenon that leads to optical property variation is structural coloration, which occurs when micro- or nanostructures reflect or scatter light, leading to constructive interference between certain wavelengths. Even though lignin is an amorphous material, surprisingly, two out of the 120 lignin samples exhibited angle-dependant surface colour, which is commonly attributed to the iridescence phenomenon. Usually, such phenomenon occurs in nanocrystals and it was already observed for nanocrystalline cellulose [52,53,54]. This could lead to new research opportunities in lignin optical properties as well as for lignin doped with compounds such as nanocrystals. 

Our future research will use principal component analysis for correlating molecular properties such as functional groups with colour and examine other extraneous factors that have an influence such as the relative size of the particles, morphology, moisture content, presence of carbohydrates and inorganic impurities. Considering the efforts required for a full chemical characterization, only 45 among the 120 in this study have been selected to be used in future work, and their colours are those illustrated in Figure 1. They have been classified based on four criteria: (a) feedstock; (b) recovery process; (c) technology readiness level; and (d) relevance for lignin users. Moreover, computer-based molecular dynamics simulations will be carried out to investigate the phenomena driving the various lignin colours at the molecular level.

## 5. Conclusions

The colour of commercial lignins is critical for their use in some high-value applications such as bioplastics, coatings, anti-oxidants, cosmetics, sunscreen additives for UV absorbance and cannot be disregarded. This work provides the first evaluation of the variability in the colours of lignins and how they can be influenced by the extraction and recovery conditions. It was observed that softwood lignins are darker than hardwood derived lignin. Also, harsh delignification such as in the Kraft pulping process results in darker lignin when compared to milder processes like organosolv. Generally, reslurrying in acidic water prior to washing allows to obtain brighter lignin than with a filtration system solely. An important result was that the stronger the mineral acid employed, the brighter the colour of the lignin produced from a given black liquor if all other conditions are kept unchanged. Furthermore, this study is one of the first steps towards promoting lignin availability from more than one source at a specified quality. It also elucidates how the lignin manufacturers can adapt their processes to target a given lignin with specific functional properties, in addition to developing robust and reliable optical soft sensors for process quality control purpose. From a quality control perspective, a supplier will need to supply lignin within the range of tolerance expected by its customers in order to sell the lignin at a specific price that is fair, representative and reflects its quality. If this cannot be achieved, a lower price can be expected and this would lead to loss of revenues. In some cases, producing outside the range of quality tolerance would necessitate post-treatment or rejection of the batch, and, subsequently additional incurred costs. We have also identified key future challenges for benchmarking commercial lignins based on colour and extending the comprehension of colour variations. Finally, we have proposed, developed and evaluated a colour analysis method that is provided in the appendix and would welcome further discussions about its use from lignin researchers.

## Figures and Tables

**Table 1:** Characteristics of black liquors.

Parameters	A	B
Total solids (%)	46.98 ± 0.19	49.00 ± 0.23
UV lignin (%)	41.94 ± 0.84	35.19 ± 0.92
Klason Lignin (% total solids)	39.24 ± 3.27	38.56 ± 3.51
Acid soluble Lignin (ASL) (mg/g)	57.01 ± 1.27	75.31 ± 1.69
Inorganics or Ash (% total solids)	55.33 ± 1.79	30.39 ± 1.86
Organics or Ash (% total solids)	44.67 ± 1.79	69.61 ± 1.86
pH at room temperature	13.10 ± 0.02	13.22 ± 0.01
Dynamic viscosity (mPa.s)	136.00 ± 2.00	119.00 ± 1.00
TOC (g/L)	186.53 ± 6.52	226.05 ± 6.97
Residual Effective Alkali (g/L as Na_2_O)	2.31 ± 0.01	23.26 ± 0.04

**Table 2:** Colours for non-carbonized and carbonized lignin and commercial carbon black.

Samples	L*	a*	b*	Δ*E*_00_ (w.r.t Carbon Black)
Commercial Lignin 1 (CO_2_)	53.59 ± 0.07	9.02 ± 0.01	25.94 ± 0.07	30.09
Commercial Lignin 2 (Dilute Acid)	29.05 ± 0.15	11.76 ± 0.05	17.39 ± 0.07	15.98
Carbonized Lignin 1	22.81 ± 0.02	0.7 ± 0.02	1.73 ± 0.03	2.91
Carbonized Lignin 2	22.86 ± 0.19	0.33 ± 0.03	0.66 ± 0.01	2.27
Reference Carbon Black (ASTM D5098)	25.77 ± 0.12	0.20 ± 0.02	−0.17 ± 0.03	0.00
Commercial Carbon Black	10.01 ± 0.10	0.33 ± 0.09	1.88 ± 0.06	10.85

## Appendix A. Method for Lignin Colour Analysis

### Appendix A.1. Introduction

Colour is the property resulting from the analysis of light by the human eye. An object’s colour is subjective since it can change with the observer and the illumination conditions. Thus, it might not be possible to make fair comparison without any standardized and reproducible approach. The purpose of this document is to specify the basis for spectral measurement and the computation of colorimetric characteristics. This test method is incorporated into this article in order to provide opinion and guidance on the analysis of lignin colour. The user agrees to credit Natural Resources Canada in any publication that involves the use of this method as obtained or with variations. Natural Resources Canada reserves the right to publish updated or newer versions of this method without prior notice. Natural Resources Canada disclaims all liabilities that may arise from the use, application or adaptations of the material published in this document.

### Appendix A.2. Scope

This Natural Resources Canada colour test method describes a systematic protocol for the analysis and quantification of lignin colour. This procedure utilizes a spectrophotometer to measure the light reflected from a lignin powder. The colour is determined by comparison of the emitted spectrum with the reflected one. The colour can be determined for lignin with high residual content (HRCL) as well as low residual content (LRCL). This method is valid for all types of lignin in particulate forms, including Kraft lignin, organosolv lignin, lignosulfonates, ionic liquid lignin, any lignin recovered from fractionated woody biomass or agricultural residues. The purpose of this present method is to be applicable and reproducible in each laboratory and industrial setting, regardless of the production quantity and without the need for high-level equipment. Methods for the correction of instrument errors and procedures for reliable visual evaluation of colour images are outside of the scope of this document.

### Appendix A.3. Normative References

The equations for computing the colour differences between a reference and other sample(s) is indispensable for applying this document and available from Sharma et al. [38].

### Appendix A.4. Terms and Definitions

#### Appendix A.4.1. Lignin

Bio-based solid matter comprising mainly of polyphenol and recovered from a biomass fractionation process as a powder.

#### Appendix A.4.2. CIE L*a*b* Colour Space

International Commission on Illumination CIE L*a*b* colour space portrays all perceivable colours in a three-dimensional mathematical space L*a*b*. The L* coordinate describes the lightness from 0 (darkest black) to 100 (brightest white). The red and green (respectively yellow and blue) opponent colours are represented along the a* dimension (respectively b*) from negative value to positive value.

#### Appendix A.4.3. Colour Difference

The colour difference allows to benchmark the distance between two colours. It is calculated based on the Euclidian distance within the colour space. The most used metrics for colour distance include correction factors since human eyes are more sensitive to certain colour nuances than devices. Since 1976, the CIE has proposed several colour difference metrics named Δ*E*. We suggest the use of CIEDE2000 (Δ*E*_00_) because it resolves the problem of perceptual uniformity, i.e., the fact that the human eyes notice more the colour difference for a blue than for a green object.

### Appendix A.5. Principle

A minimum quantity of 25 g of lignin sample is dried at a low temperature ranging from 55 to 65 °C. The dry sample is then placed in an optically clear glass cup and filled to a predetermined volume. A good quality plastic and optically clear cup could also be used. As scratches or stains often occur on the surfaces of plastic cup, it is recommended to use optical quality glass cup. Short after, the colour measurements are performed with a spectrophotometer using the CIE L*a*b* colour scale. The differences between a reference colour and the measurements are also calculated.

### Appendix A.6. Equipment and Reagents

- Aluminium pans
- Measuring cylinder
- Drying oven
- Desiccator, loaded with a desiccant
- Optically clear glass cup made from fused silica with a 25 mL mark
- Spectrophotometer with a 45°/0° measurement geometry, and a 65/10 illuminant/observer.

### Appendix A.7. Sampling

Although sampling methods are outside the scope of this method, adequate care should be taken to ensure that the golden rules for powder sampling are respected and that samples used are representative of the bulk from which they are obtained.

### Appendix A.8. Procedure

- Samples are to be analysed in triplicate with the mean result and the standard deviations presented
- Weigh the aluminium pan to the nearest 1 mg
- Add sufficient dry lignin samples to cover to fill the clear glass cup up to the 25 mL mark
- Place the sample in an oven and re-dry it at 55 °C ± 5 °C for at least 2 h until a constant weight is reached or a maximum duration of 24 h
- Remove the sample from the oven and allow it to cool down to room temperature in a desiccator
- Calibrate the spectrophotometer as per the manufacturer’s instruction
- Fill the glass sample cup with the sample up to the 25 mL mark
- Select the CIE L*a*b* colour scale for all measurements
- Cover the sample with an opaque cup to prevent interference by external light sources and place on or in the measurement port
- Perform measurement in triplicate.

### Appendix A.9. Calculation

Any of the lignin powders can be selected as a reference and the colour variations between the samples can be quantified from the coordinates with the CIEDE2000 (Δ*E*_00_) formula below, with parameters and derivations for computation as given in [37,38,39]:

(A1) $$\Delta E_{00}=\sqrt{{\left(\frac{\Delta L^{\prime }}{k_{L}S_{L}}\right)}^{2}+\;{\left(\frac{\Delta C^{\prime }}{k_{c}S_{c}}\right)}^{2}+\;{\left(\frac{\Delta H^{\prime }}{k_{H}S_{H}}\right)}^{2}+R_{T}\frac{\Delta C^{\prime }}{k_{C}S_{C}}\frac{\Delta H^{\prime }}{k_{H}S_{H}}}.$$

In addition, the Browning Index (*BI*) for all lignin samples can be quantified with the equation:

(A2) $$BI=100\;\times \;\left(\frac{X-0.31}{0.17}\right),$$

where:

(A3) $$X=\frac{\left(a^{*}+1.75L^{*}\right)a^{*}}{\left(5.645L^{*}+\;a^{*}-3.012b^{*}\right)}.$$

### Appendix A.10. Report

Any communications or publication shall refer to this Natural Resources Canada colour test method. In addition, the following shall be included for a test report:

- Date and place of testing;
- Identification of the samples tested;
- Results expressed as the CIE L*a*b* colour coordinates for a sample as well as the range and standard deviation;
- Any variation from this procedure as described in the Natural Resources Canada colour test method and/or any circumstance that might have had an impact on the results.

### Appendix A.11. Precision Repeatability

Any communications analysis of softwood Kraft lignin was determined using the method described above. The average of triplicate determinations is given in Table A1.

**Table A1:** CIE L*a*b* coordinates for softwood Kraft lignin (*n* = 3).

Sample	L*	a*	b*
Softwood Kraft Lignin	53.590	9.020	25.940
Standard Deviation	0.068	0.012	0.068
Range	0.136	0.023	0.136
